# Unexpected curative effect of PD-1 inhibitor in gastric cancer with brain metastasis: A case report

**DOI:** 10.3389/fonc.2023.1042417

**Published:** 2023-02-16

**Authors:** Qijun Wang, Zhewei Shen, Mengxi Ge, Jie Xu, Xin Zhang, Wei Zhu, Jie Liu, Wei Hua, Ying Mao

**Affiliations:** ^1^ Department of Neurosurgery, Huashan Hospital, Fudan University, Shanghai, China; ^2^ National Center for Neurological Disorders, Shanghai, China; ^3^ Shanghai Key Laboratory of Brain Function Restoration and Neural Regeneration, Shanghai, China; ^4^ Neurosurgical Institute of Fudan University, Shanghai, China; ^5^ Shanghai Clinical Medical Center of Neurosurgery, Shanghai, China; ^6^ Department of Oncology, Huashan Hospital, Fudan University, Shanghai, China; ^7^ Institute of Biomedical Sciences, Fudan University, Shanghai, China; ^8^ Department of Digestive Diseases, Huashan Hospital, Fudan University, Shanghai, China

**Keywords:** brain metastasis, gastric cancer, PD-1/PD-L1, PD-1 inhibitors, immunotherapy

## Abstract

**Background:**

Gastric cancer (GC) is the third most common cause of cancer-related death in the world. Several clinical trials have proven that the use of PD-1/PD-L1 inhibitors can improve the survival of late-stage GC patients and is suggested in NCCN and CSCO guidelines. However, the correlation between PD-L1 expression and the response to PD-1/PD-L1 inhibitors is still controversial. GC rarely develops brain metastasis (BrM) and currently there is no therapeutic protocol for GC BrMs.

**Case presentation:**

We report a case of a 46-year-old male suffering from GC with PD-L1 negative BrMs 12 years after GC resection and 5 cycles of chemotherapy. We treated the patient with the immune checkpoint inhibitor (ICI) pembrolizumab and all metastatic tumors achieved a complete response (CR). A durable remission of the tumors is confirmed after 4 years of follow-up.

**Conclusion:**

We shared a rare case with PD-L1 negative GC BrM responsive to PD-1/PD-L1 inhibitors, the mechanism of which is still unclear. The protocol of therapeutic choice for late-stage GC with BrM is urgently needed. And we are expecting biomarkers other than PD-L1 expressions to predict the efficacy of ICI treatment.

## 1 Introduction

Brain metastasis (BrM) is one of the most common brain tumors in adults (1). An estimate of 20% of patients with cancer will suffer from BrMs (1). Lung cancer, breast cancer, and melanoma are the most frequent primary sites to develop BrMs. GC rarely develops BrMs and only accounts for 6% of all BrMs (2). However, in China, GC ranks the second most frequently reported case among all malignant tumors, just behind lung cancer, and most of them are diagnosed in their late stage, which gives GC more opportunities to spread to distant organs (3). Traditionally, medical practices for BrMs include surgery combined with chemo- and radio-therapy. As for immunotherapy, high expression of PD-L1 is associated with poor prognosis in GC (4). Although the use of PD-1 inhibitor in GC has been included in 2022 NCCN guidelines and approved to be effective (5), the efficacy of PD-1 inhibitors against GC with BrM is not confirmed yet. In this case report, a GC patient with PD-L1 negative BrM was treated with pembrolizumab and later achieved an amazing recovery. Our results showed the possible efficacy of PD-1/PD-L1 inhibition for GC BrM patients, even in PD-L1 negative cases.

## 2 Case report

**Figure 1:**
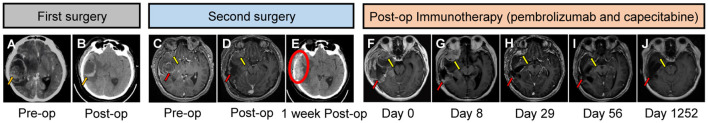
The timeline and brain imaging over course of treatment. Enhanced computed tomography (CT) image **(A)** showed a right temporal lesion (orange arrow) before the first cranial surgery. The lesion was resected as shown in figure **(B)** (orange arrow). Magnetic resonance imaging (MRI) (T1-weighted with gadolinium enhancement) **(C)** demonstrated a recurrent right temporal lesion (red arrow) *in situ* and a right frontal tumor (yellow arrow). MR T1 contrast **(D)** demonstrated the resected space of the right temporal tumor (red arrow) after the second cranial surgery. CT scanning **(E)** indicated an extradural hematoma (red circle) one week after the second cranial surgery. Figure **(F–J)** represent the MR T1 contrast images 1 day, 8 days, 29 days, 56 days and 1252 days after pembrolizumab administration.

**Figure 2:**
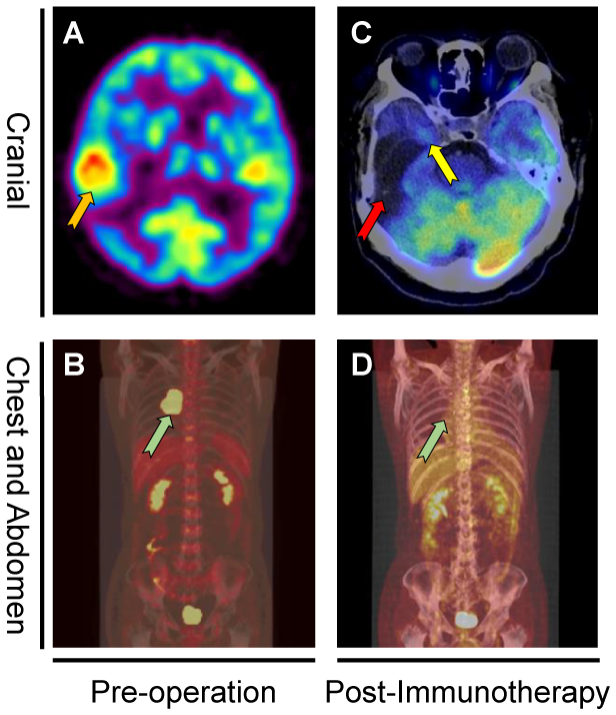
PET-CT before first cranial surgery and after immunotherapy. PET-CT showed a right temporal lesion (orange arrow) before the first cranial surgery **(A)**. Coronal PET-CT image **(B)** of the chest showed a right mediastinal metastasis (green arrow). Figure **(C)** showed the original tumor sites (red arrow and yellow arrow) remained stable without diseases recurrence. Coronal PET-CT image **(D)** of the chest indicated the tumor site (green arrow) remained stable without recurrence 16 months after mediastinal surgery.

The patient is a 46-year-old male who received surgery and 5 cycles of chemotherapy for GC 12 years ago. In September 2018, the patient has suffered from severe headache with nausea and vomiting for one week and was admitted to our hospital. During the physical examination, the patient presented a positive Babinski sign and a grade IV muscle strength on the left side. Head computed tomography (CT) demonstrated a right temporal mass (**Figure 1A**). Positron-emission tomography-computed tomography (PET-CT) of the brain (**Figure 2A**) and chest (**Figure 2B**) showed evidence of multiple metastases.

**Figure 3:**
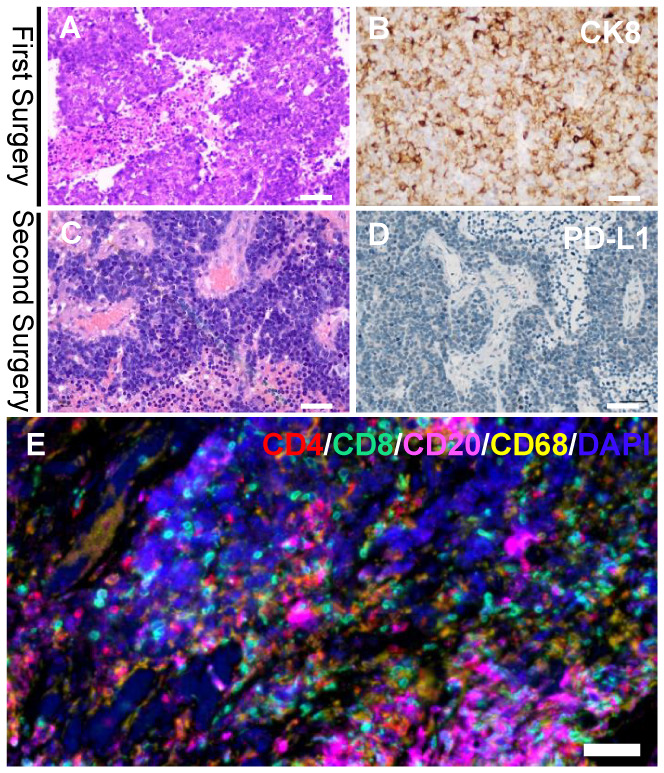
Histologic findings of the first and second cranial surgery. Hematoxylin and eosin (HE) staining **(A)** and positive cytokeratin 8 (CK8) immunohistochemistry staining **(B)** of the tumor tissue indicated a poorly differentiated adenocarcinoma (magnification, bar=50μm). HE staining **(C)** of the tumor tissue demonstrated a recurrent adenocarcinoma (magnification, bar=50μm). Immunohistochemistry of PD-L1 **(D)** was completely negative in both immune cells and tumor cells (magnification, bar=50μm). Multiplex immunohistochemistry/immunofluorescence (mIHC/IF) assay of the second brain metastasis specimens combining CD4, CD8, CD20 and CD68 markers **(E)** displayed a dense tumor-infiltrating CD8+ T lymphocytes surrounding the tumor cells (magnification, bar=50μm).

In October 2018, the patient underwent his first cranial surgery (**Figure 1B**). Post-operative pathological test confirmed a metastatic adenoma (**Figures 3A, B**). One month later in November 2018, the patient underwent another surgery to remove the mediastinal metastasis in another hospital instead of administrating systemic chemotherapy. In December 2018, the patient was admitted to our emergency room once again with severe headache. Head MRI (**Figure 1C**) demonstrated a recurrent right temporal tumor (red arrow) *in situ* and a newly diagnosed right frontal tumor (yellow arrow) with apoplexy. A second emergency cranial surgery was performed. The right temporal tumor was resected, and the right frontal tumor was too risky to resect under the same bone window then left for chemo- and targeted therapy (**Figure 1D**). Unfortunately, one week after the second surgery, the patient suffered from severe headaches. CT showed an extradural hematoma in the surgical field (**Figure 1E**, red circle). However, the patient’s relatives declined further surgical intervention. After systemic mannitol and intracranial pressure control, the hematoma remained stable.

Hematoxylin and eosin (HE)-stained (**Figure 3C**) specimens of the brain lesion revealed a poorly differentiated adenocarcinoma. Immunohistochemical (IHC) staining (**Figure 3D**) revealed the BrM was completely negative for B7H1 (PD-L1) in both immune cells and tumor cells, as the combined positive score (CPS) and tumor cell proportion score (TPS) of PD-L1 in this patient was rated below 1 and 1% respectively. However, we didn’t know if PD-1 inhibitors would work in PD-L1 negative BrM patients. Therefore, in January 2019, pembrolizumab (200mg, iv. Day1) plus capecitabine (1.5g, bid, po. Day1-14) were still tried to treat the right frontal tumor (**Figure 1F**) based on the advice from a neuro-oncologist. To our surprise, the response was remarkable. Four weeks after the first cycle of administration, the MRI (**Figure 1H**) showed that the tumor shrank nearly 80% compared to 3 weeks earlier (**Figure 1G**). The patient then received 10 cycles of pembrolizumab plus capecitabine therapy since February 2019 and recovered quite well without any side effects. Eight weeks after the treatment began, the tumor site achieved a complete response (CR) (**Figure 1I**) according to the Response Evaluation Criteria in Solid Tumors (Recist, version 1.1). In March 2020, another PET-CT image confirmed a durable remission of the tumors in both head and chest (**Figures 2C, D**). During the last follow-up (May 2022), the MRI indicated no abnormal signal in the surgical field and right frontal lobe (**Figure 1J**). The overall clinical course is shown in **Figure 1**.

## 3 Discussion

Immunotherapy salvaged this late-stage GC patient, as the immune reaction against tumors was ignited by the PD-1 inhibitor. It has been reported that PD-L1 was highly expressed in GC and showed a negative correlation with survival (6). Given the encouraging results of immune checkpoint inhibitors (ICIs) in melanoma, non-small-cell lung cancer, renal cell cancer, and head and neck cancer, it seems reasonable to investigate the curative effect of these agents in GC.

Avelumab, a human anti-PD-L1 IgG1 antibody, has had its efficacy and safety validated in a phase Ib trial of advanced gastric or gastroesophageal junction adenocarcinoma patients (7). The results showed the objective response rate (ORR) was 6.7% in both first-line switch-maintenance (1L-mn) and second-line (2L) subgroups and the median OS was 11.1 and 6.6 weeks in two arms (1L-mn and 2L respectively), with a nonsignificant trend toward longer OS for PD-L1-positive patients in the 1L-mn subgroup (7). Durvalumab, another anti-PD-L1 antibody, showed clinical activity in GC with an ORR of 7% in a phase I study (8). Meanwhile, the phase I clinical trial of atezolizumab (anti-PD-L1 antibody), including 175 advanced incurable patients, confirmed complete and partial responses in 21% of non-small-cell lung cancer, 26% of melanoma, and 13% of other tumors including GC and colorectal cancer (9). And a statistical association between treatment response and PD-L1 expression in tumor-infiltrating immune cells was observed (9).

It is believed that the blood-brain barrier (BBB) is disrupted in BrM cases, resulting in a ‘leakier’ condition called blood-tumor barrier (BTB) (10), allowing ICIs to pass through and accumulate within the brain. Therefore, some BrMs could also benefit from ICI treatment. In a phase II trial of pembrolizumab in 18 melanoma and 18 non-small cell lung cancer (NSCLC) patients, the durable response rate of intracranial metastasis was 22% and 33%, respectively (11). In another cohort of 66 melanoma patients with BrMs treated with nivolumab or pembrolizumab, the intracranial ORR was 21% (12).

Traditionally, a low PD-L1 expression is believed to be associated with shorter progression free survival (PFS) and lower ORR (13, 14). Why is this PD-L1 negative GC BrM patient sensitive to anti-PD-1/PD-L1 immunotherapy? One reason might be that PD-L1 expression status alone is insufficient in determining which patients could benefit from PD-1/PD-L1 inhibitors (15). In addition, PD-L1 expression is heterogenous between primary tumor, metastatic lymph nodes and the BrM (16). Therefore, the drug responses would be determined by the integral PD-L1 expression status. As it is now impossible to know the PD-L1 expression rate in the primary tumor site of this patient, the validity of this explanation might be hard to prove. Besides, it is also reported that chemotherapy and immunotherapy together may reengineer tumor immunity and harness potential synergies (17, 18), which may happen in this case.

The BrM patients responsive to PD-1 inhibitor might get a dramatic clinical relief (11, 12). However, the response ratio remains quite low (9), making it essential to pick the right population. To delineate the mechanism behind this rare case, we performed a multiplex immunohistochemistry/immunofluorescence (mIHC/IF) assay combining CD4, CD8, CD20 and CD68 markers on the second cranial metastasis surgery specimens (**Figure 3E**) to show the pre-existing T cells, which are believed to be the prerequisite to the anti-PD-L1 response (19). The specimen displayed a dense tumor-infiltrating CD8+ T lymphocytes accompanied by CD68+ macrophages, which demonstrated an intense antitumor immune response within the tumor microenvironment and indicated a positive response to anti–PD-1/PD-L1 treatment.

In the context of immunotherapy, ICI treatment has been widely used in metastatic cancers other than GC BrM (9). Like primary solid tumors, abundant infiltrating T cells in the tumor microenvironment (TME) is the basis of a successful ICI treatment in disseminated cancers (20). Biomarkers associated with PD-1/PD-L1 treatment should be further explored to direct a more precise ICI treatment in metastatic cancers. In clinical scenario which needs rescue therapy like this case, ICI treatment is always in consideration regardless of PD-L1 expression unless a more convincible biomarker shows unfavorable result.

In summary, we have presented a case of PD-L1 negative GC BrM. After pembrolizumab treatment, all metastatic tumors achieved a surprisingly complete response. Our findings have proven that PD-L1 expression is not the only determinant of ICI responsiveness. Other biomarkers are urgently needed.

## Data availability statement

The raw data supporting the conclusions of this article will be made available by the authors, without undue reservation.

## Ethics statement

Written informed consent was obtained from the individual(s) for the publication of any potentially identifiable images or data included in this article.

## Author contributions

QW and ZS completed the main body of the manuscript. YM, WZ, WH, MG, XZ, JL and JX made decisions about the entire treatment process. YM and WH have critically revised the manuscript. ZS, QW and WH participated in the collection of patient data. All authors contributed to the article and approved the submitted version.
